# Pilot clinical trial of neoadjuvant toll-like receptor 7 agonist (Imiquimod) immunotherapy in early-stage oral squamous cell carcinoma

**DOI:** 10.3389/fimmu.2025.1530262

**Published:** 2025-01-27

**Authors:** Angela J. Yoon, Richard D. Carvajal, Evan M. Graboyes, John M. Kaczmar, William G. Albergotti, Alexandra E. Kejner, Scott H. Troob, Elizabeth Philipone, Jean-Sebastien Anoma, Kent E. Armeson, Elizabeth G. Hill, Mary S. Richardson, Tina R. Woods, Bhishamjit S. Chera, Farzad Nourollah-Zadeh, Byung J. Lee, Subramanya Pandruvada, Antonis Kourtidis, Christina Kingsley, Elizabeth C. O’Quinn, Stephanie Mills, Victoria C. Jordan, Mike Spencer, Danielle Fails, Trevor D. McKee, Mark Zaidi, Alan Brisendine, Shane Horn, Shikhar Mehrotra, Besim Ogretmen, Jason G. Newman

**Affiliations:** ^1^ Medical University of South Carolina, Charleston, SC, United States; ^2^ Northwell Health Cancer Institute, New Hyde Park, NY, United States; ^3^ Columbia University Irving Medical Center, New York, NY, United States; ^4^ Fortis Life Sciences, Montgomery, TX, United States; ^5^ Pathomics, Toronto, ON, Canada

**Keywords:** oral cancer, neoadjuvant clinical trial, toll-like 7 receptor agonist, imiquimod, immunotherapy

## Abstract

**Background:**

There is no neoadjuvant immunotherapy for early-stage oral cancer patients. We report a single-arm, open-label, pilot clinical trial assessing the efficacy and safety of topical toll-like receptor-7 (TLR-7) agonist, imiquimod, utilized in a neoadjuvant setting in early-stage oral squamous cell carcinoma (OSCC).

**Methods:**

The primary endpoint is reduction in tumor cell counts assessed by quantitative multiplex immunofluorescence and the immune-related pathologic response. The secondary endpoint is safety.

**Results:**

60% of patients experienced a 50% reduction or greater in tumor cell count post-treatment (95% CI = 32% to 84%). Similarly, 60% of patients had immune-related major pathologic response (irMPR) with two complete pathologic responses, and 40% had partial response (PR) with the percent residual viable tumor ranging from 25% to 65%. An increase in functional helper and cytotoxic T-cells significantly contributed to a reduction in tumor (R=0.54 and 0.55, respectively). The treatment was well tolerated with the application site mucositis being the most common adverse event (grades 1-3), and no grade 4 life-threatening event. The median follow-up time was 17 months (95% CI = 16 months - not reached), and one-year recurrence-free survival was 93% of evaluable patients.

**Conclusion:**

Neoadjuvant imiquimod immunotherapy could be safe and promising regimen for early-stage oral cancer.

**Trial registration:**

ClinicalTrials.gov, Identifier NCT04883645.

## Introduction

Immunotherapy in a neoadjuvant setting is a promising approach to complement definitive surgery in head and neck cancer (1–3). The neoadjuvant use of immunotherapy as opposed to adjuvant setting is associated with improved clinical outcomes due to the stronger antitumor immune response generated in the presence of tumor (4, 5). We conducted a pilot clinical trial using immunomodulatory agent, toll-like receptor 7 (TLR)-7 agonist (imiquimod 5% cream) pre-operatively. Imiquimod exerts an anti-tumor effect by stimulating innate and adaptive immunity (6–11). Moreover, imiquimod increases tumor cell apoptosis by shifting the proapoptotic and antiapoptotic Bcl factors toward the proapoptotic Bax protein, and by stimulating the release of mitochondrial cytochrome c into the cytosol, activating caspase-9 and caspase-3 (6–11).

Oral squamous cell carcinoma (OSCC), the most common type of head and neck cancer, is typically treated with surgical resection followed by irradiation or chemotherapy if indicated (12–15). In recent years, immunotherapy has emerged as a promising therapeutic option in treating head and neck cancer (16). OSCC harbor an anatomical advantage compared to other solid tumors, as they are easily accessible to physical examination owing to direct accessibility and visibility, allowing for self-application of drugs and real-time monitoring without the need for imaging studies (2, 6). Hence, topical treatment with imiquimod in the neoadjuvant setting is an attractive therapeutic strategy for patients with early-stage OSCC. Direct application of therapy agent maximizes local bioavailability while ensuring minimal systemic toxicity. Imiquimod absorbed through mucosa primes and initiates an antitumor immune response, contributing to improved clinical outcomes (17, 18).

We performed an open-label, single-agent pilot study to evaluate neoadjuvant topical imiquimod’s antitumor activity and safety in patients with early-stage OSCC. We also assessed a shift in the tumor microenvironment (TME) immune profile, PD-L1 expression-based biomarker, and one-year recurrence-free survival. This is the first neoadjuvant imiquimod clinical trial in early-stage oral cancer patients.

## Patients and methods

### Patients

Eligible patients had newly diagnosed and histology-confirmed OSCC, with clinical T1 or T2 (American Joint Committee on Cancer, 8^th^ Ed [AJCC 8]) without nodal involvement (N0) and distant metastases (M0) assessed by imaging studies (TNM Stages I and II), and Eastern Cooperative Oncology Group (ECOG) Performance Status ≤2. The patients were treatment-naïve and planned for surgical resection with curative intent.

### Trial design

This was a single-arm, open-label pilot trial to assess the feasibility, efficacy, and safety of utilizing imiquimod 5% cream applied topically in a neoadjuvant setting for 28 days immediately followed by surgical resection of the tumor. The trial was conducted following the protocol approval from the Institutional Review Boards. Patients were enrolled at Columbia University Irving Medical Center (CUIMC) in New York, NY from September 2021 to February 2022, and at Medical University of South Carolina (MUSC) in Charleston, SC from May 2022 to September 2023.

Topical imiquimod 5% cream was self-administered starting day 1 of a 28-day cycle. Patients applied the cream directly onto the tumor and the surrounding area at bedtime, left it on for 20 minutes, and rinsed thoroughly. Self-application of imiquimod was performed daily for 28 days, and the date and time of the application were documented in the Daily Diary form. Clinical tumor size was measured at the baseline, midpoint (14 days into the trial), and post-therapy by measuring the longest perpendicular bidirectional size of the clinically visible lesion. The adverse events (AEs) were recorded and graded for safety evaluation according to the NCI Common Terminology Criteria for Adverse Events (CTCAE version 5.0) throughout the treatment period.

The primary endpoint was a minimum of 50% reduction in tumor cell count assessed by quantitative multiplex immunofluorescence (qmIF) within the tumor bed of the surgical tissue (post-treatment) compared to the biopsy tissue (pre-treatment). In addition, the major pathologic response was assessed using Immune-Related Pathologic Response Criteria (irPCR) in the tumor bed of the post-treatment surgical tissue. The secondary endpoint was treatment-related toxicity graded by CTCAE, defined as safe if no life-threatening (Grade 4) is reported. The correlative endpoint was a shift in antitumor immune profile in post-treatment tissue compared to pre-treatment tissue measured by qmIF. PD-L1 expression was evaluated on tumor and tumor-infiltrating immune cells to assess the combined positivity score (CPS). The electronic chart review was conducted every 12 weeks to determine recurrence and survival status. The one-year recurrence-free survival (RFS) was measured from the time of surgery to the time of biopsy-confirmed recurrent OSCC.

The study was approved by the Institutional Review Board and conducted in accordance with institutional and federal guidelines for human investigation in accordance with the Declaration of Helsinki. All participants were informed of the investigational nature of the study and provided written informed consent prior to enrollment. Ongoing safety oversight was conducted by the Institutional Review Board and Safety Monitoring Committee.

### Pathologic analyses

Immune-related Pathologic Response Criteria were used to assess the pathologic response to immunotherapy (19–21). The H&E stained slides were prepared from formalin-fixed, paraffin-embedded surgical specimens. Two board-certified pathologists blinded to patient outcome scored the slides by identifying the areas of tumor bed and residual viable tumor. The tumor bed was defined as the sum of 1) residual viable tumor (RVT), 2) necrosis, and 3) regression bed characterized by the presence of dense tumor-infiltrating lymphocytes (TIL) infiltrates, tertiary lymphoid structure (TLS), features of cell death (keratinous debris and giant cells), and tissue repair (neovascularization, fibrosis). The %irRVT was calculated as RVT area/tumor bed area x 100. If the estimate of %irRVT differed by >10% between two pathologists, a third pathologist served as an adjudicator to finalize the score. pCR (complete response) was defined as 0% irRVT, irMPR (major pathologic response) as <10% irRVT, PR (partial response) as >10 to 90% irRVT, and irNR (no response) as >90% RVT.

### Quantification of tumor and immune cells

Tissues were macrodissected to separate the tumor bed from the overlying surface epithelium and surrounding stroma in the biopsy and surgical tissues by a pathologist blinded to the pre- and post-treatment samples. Tumor and immune cells in the TME were quantified using qmIF. The slides were stained with pan-cytokeratin using Lunaphore Comet automated stainer (Fortis Life Sciences, TX) for quantification of pan-cytokeratin (pan-CK) positive tumor cells (22). In addition, tumor cells were assessed for proliferating cell nuclear antigen (PCNA) expression. Similarly, the immune profile between the pre- and post-treatment TME was compared using qmIF. To characterize immune cells, the markers for CD3 (T-cells), FoxP3 (Regulatory T-cells), CD4 (Helper T-cells), CD8 (Cytotoxic T-cells), PD-L1 (Immune response co-inhibitory factors on tumor and macrophages), CD45 (Lymphocytes), CD45RO (memory T-cells), Granzyme B (T-cell activation), CD68 (Macrophages) were used.

The digital image analysis and quantification, segmentation, and classification of immunofluorescence-positive cells were meticulously overseen by a pathologist, adhering to the methodology previously outlined (23). Initially, images were imported into the QuPath (RRID: SCR_018257) version 0.3.2. Cellular segmentation was executed using the StarDist algorithm. To ensure accuracy, marker-specific thresholds were established using a composite image that encapsulated representative samples from the entire image batch. These individual thresholds were amalgamated to form a composite classifier, enabling the categorization of each cell into distinct classes based on marker positivity. Within 6-8 regions of interest (ROI), quantitative analyses were conducted to enumerate the cells corresponding to each marker or combination thereof. This count was then standardized against the total area of the tissue region under examination, thereby deriving cell-type density metrics and expressed as cells/mm^2^.

### Spatial score analysis

Spatial Score was calculated to generate mean spatial ratios per sample between permutations of sets of 3 cells (26, 27). The X/Y coordinates for each cell type were determined during cellular segmentation. The minimal distance between each cell type and its nearest other cell types and the averages of these minimal distances per tissue spot were calculated in a python script adapted from the original R script (github.com/nolanlab/SpatialScore). To assess the relationship of cell distances between three cell types [Tumor cell (C1), T-cell (C2), and Macrophage (C3)], the minimal distances between CT1-CT2 (left distance) versus CT2-CT3 (right distance) were calculated. This distance ratio (Spatial Score) was determined as left distance/right distance. The median ratio from all samples was compared between pre- and post-treatment.

### Assessment of PD-L1 expression

The tumor proportion score (TPS), which measures the proportion of residual viable tumor cells expressing PD-L1, and the combined positive score (CPS), which measures the proportion of PD-L1 on both tumor cells and macrophages, were assessed using qmIF (24, 25). CPS was defined as the proportion of PD-L1+ tumor cells divided by the sum of PD-L1+ tumor cells and PD-L1+ immune cells. The TPS cutoff values were 1% and 50%. The threshold for CPS was <1%, 1-9%, 10-19%, and >20%.

### Statistical analysis

Based on the sample size calculation, with 15 subjects and an estimated response rate of 0.5, we would achieve the 95% confidence interval (CI) of 0.239 to 0.761, using a two-sided exact binomial test with a type I error of 0.05. Baseline characteristics were summarized using median and range for continuous variables and frequency and percent for categorical variables. For tumor size, we reported median percent change from pre- to post-therapy and tested the significance of the change from baseline using the Wilcoxon signed rank test with continuity correction. The mean pre- and post-treatment tumor cell count/mm^2^ is reported with standard deviation, and the significance of differences in cell counts was assessed using the Wilcoxon signed rank exact test and Wilcoxon signed rank test with continuity correction. The proportion of patients experiencing a reduction in tumor cell count/mm^2^ from pre- to post-therapy of at least 50% was summarized as a percentage and an exact binomial 95% confidence interval. The percentage of patients with irMPR, PR, and irNR in surgical specimens was reported with the range. The significance of the change in cell counts for immune markers comparing post-therapy to pre-therapy was performed using Wilcoxon signed rank test with continuity correction as needed. The PD-L1 expression assessed in terms of TPS and CPS, and the spatial ratio between macrophages, T-cells, and tumor cells are reported in descriptive summaries. Recurrence-free survival (RFS) was defined as the time from surgery to disease recurrence/death due to any cause or date of last follow-up, whichever occurred first. RFS was censored for patients free of recurrence at the last date of follow-up. Only patients with a recurrence or minimum follow-up of 1 year were evaluated and included in the one-year RFS analysis. Due to only one recurrence among 14 RFS-evaluable patients, analysis was limited to descriptive summaries. The median follow-up time for the evaluation of RFS was calculated using the reverse Kaplan-Meier (KM) method. All analyses were performed using R version 4.3.0. All hypothesis tests were two-sided, and *p*-values < 0.05 were considered statistically significant.

## Results

### Patients and demographics

A total of sixteen patients with new primary early-stage oral cancer were enrolled from 2021 to 2023, and fifteen patients received trial therapy and were evaluated for safety and anti-tumoral response. One patient with a history of myocardial infarction had sudden cardiac death less than two weeks into the trial and was subsequently excluded from analyses. All 15 patients underwent surgical resection within two weeks of imiquimod therapy completion. The patient characteristics of fifteen evaluable patients are in **Table 1**.

**Table 1:** Baseline characteristics of the 15 study patients.

Characteristics (n=15)	Counts (%)
Age, median (range)	65 (52-87)
Gender	
Female	6 (40%)
Male	9 (60%)
Race	
White	12 (80%)
Black	2 (13%)
Asian	1 (7%)
Ethnicity	
Non-Hispanic/Latino	14 (93.%)
Hispanic or Latino	1 (7%)
ECOG performance status score	
0	15 (100%)
1	0
2	0
Smoker	
No	7 (47%)
Former, <10 pack-year	5 (33%)
Former, > 10 pack-year	2 (13%)
Current	1 (7%)
Tumor site	
Tongue (lateral and/or ventral)	9 (60%)
Floor of mouth	2 (13%)
Palate (soft and/or hard palate)	2 (13%)
Gingiva/alveolar mucosa	2 (13%)
Histologic grade	
Well-differentiated	7 (47%)
Moderate- to poorly-differentiated	8 (53%)
Clinical TNM Stage^a^	
I	10 (67%)
II	5 (33%)

^a^ *American Joint Committee on Cancer, 8^th^ Edition staging*.

### Safety and tolerability

Adverse events (AEs), at least possibly related to the study treatment, occurred in 14 out of 15 patients. **Table 2** summarizes the number of patients with treatment-related toxicities by NCI CTCAE grade. The most common therapy-related AEs were application site mucositis and discomfort. Short dose interruption (<10% of total treatment duration) occurred in two patients (13%) due to toxicity interfering with oral intake. Two patients (13%) reported fatigue during the first two weeks following the start of the study, with subsequent resolutions. No life-threatening (grade 4) AEs were reported. All patients completed the imiquimod therapy and received scheduled surgery. AEs reported by individual participants are shown in **Supplementary Table 1**.

**Table 2:** Therapy-related adverse events experienced in fourteen of fifteen patients assessed by NCI Common Terminology Criteria for Adverse Events Version 5.0 (CTCAE) grade.

Adverse Event Grade	Patients (n=15)			
	Any	1	2	3
Oral mucositis	12	5	6	1
Oral pain/Sore throat	6	2	3	1
Fatigue	2	1	1	
Oral hemorrhage	1		1	
Nausea	1	1		

CTCAE Grades:

1= asymptomatic or mild

2= moderate, interventions indicated

3= severe, interfering with oral intake

### Antitumor activity

**Figure 1:**
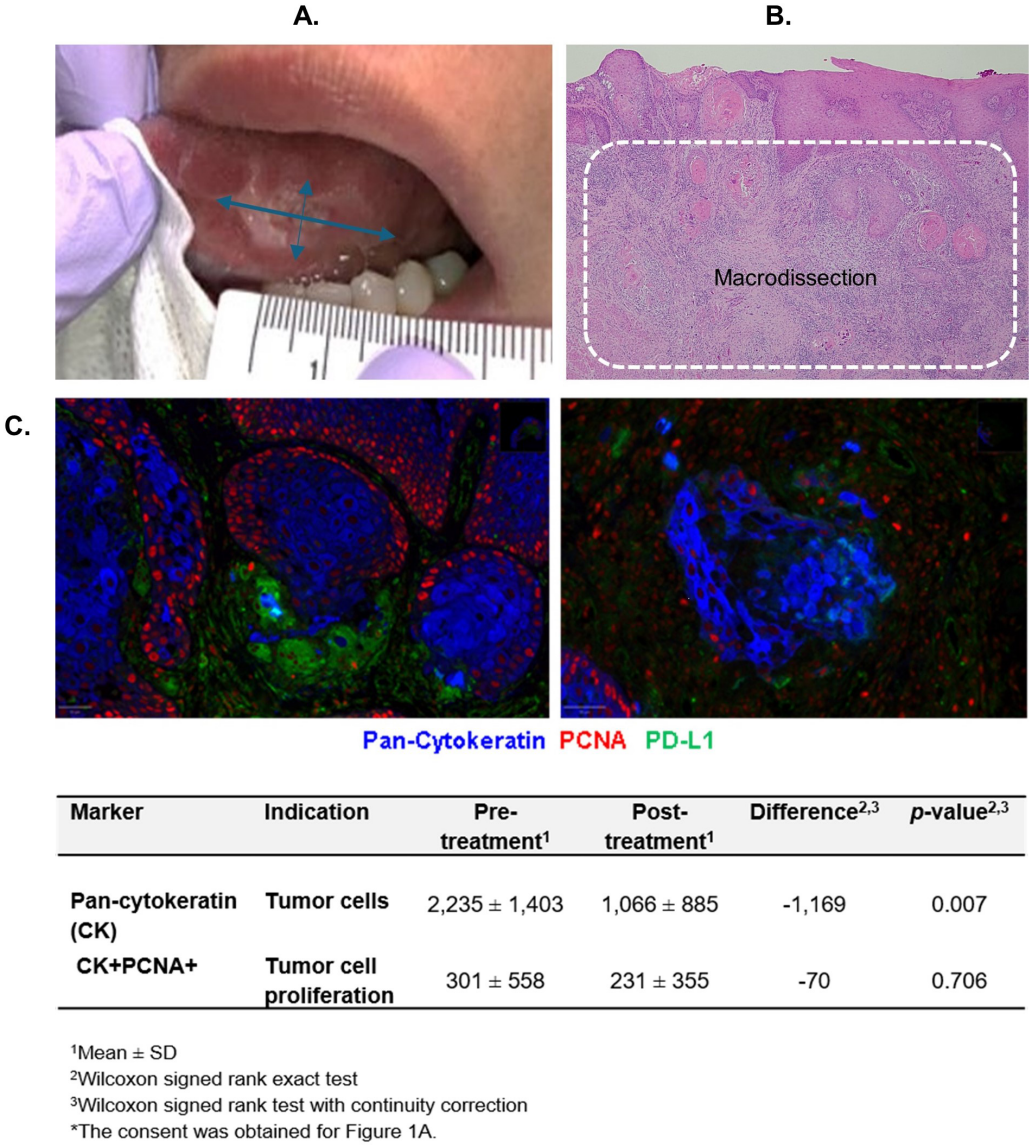
Measure of antitumor activity. **(A)** Clinical tumor size measurement. **(B)** Tumor bed area macrodissected for quantitative multiplex immunofluorescence (qmIF) analyses. **(C)** Tumor cell count reduction post-treatment (right) compared to pre-treatment (left) assessed by qmIF in 1 mm^2^ (cells/ mm^2^).

A median of 45% reduction in clinical size (IQR = -55, -28; *p*=0.001) was noted. The clinical tumor size was measured by examining the longest perpendicular bidirectional dimensions (**Figure 1A**). Fourteen (93%) patients experienced a regression in clinical tumor size after the treatment. One patient had no change in the overall size. However, the rough and irregular surface of the exophytic tumor at baseline regressed to a nearly flat lesion by the endpoint (**Supplementary Figures 1A-F**).

The tumor cell numbers were quantified using quantitative multiplex immunofluorescence (qmIF) from the tumor bed macrodissected from the biopsy and surgical tissues (**Figure 1B**). A significant reduction in tumor cell count was observed post-treatment compared to pre-treatment (*p*=0.007) (**Figure 1C**). Nine (60%) patients experienced a >50% reduction in tumor cell count after the treatment (95% CI = 32% to 84%). The baseline expression of PCNA on tumor cells was low. An insignificant decrease in PCNA expression was observed after the treatment.

**Figure 2:**
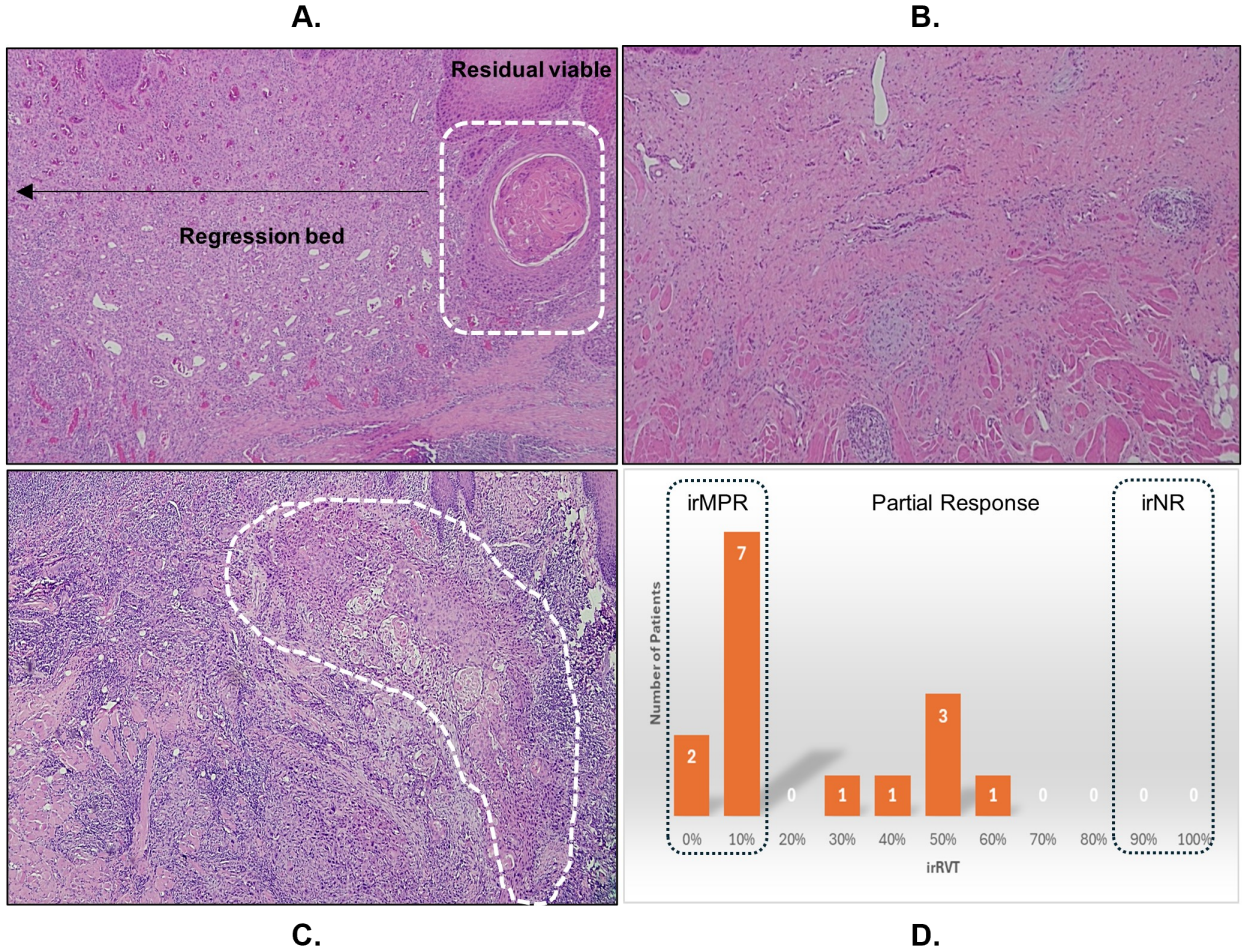
The measure of immune-mediated antitumor activity by **(A)** assessing the residual viable tumor (irRVT; white dashed line) in the tumor bed of post-treatment surgical specimen, showing major pathologic response with ≤10% irRVT. **(B)** Pathologic complete response. **(C)** Partial response (>10-90% irRVT). **(D)** Summary irRVT of all study participants.

The pathologic response in terms of irRVT ranged from 0 to 65%, with a median of 10% irRVT. The tumor regression bed was characterized by inflammatory infiltrates, keratin debris surrounded by histiocytes, neovascularization, and fibrosis. None of the cases exhibited necrosis. Based on the analysis of surgical specimen using irPRC, nine patients (60%) had irMPR (**Figure 2A**), two (13%) of whom had a complete pathologic response with no residual tumor (**Figure 2B**). Six patients (40%) had PR (**Figure 2C**). All patients showed pathologic response to immunotherapy (no irNR), as illustrated in **Figure 2D**. The change in clinical tumor size, tumor cell count, %irRVT, and pathologic response in terms of pCR, irMPR, PR, and irNR for individual patients is summarized in **Supplementary Table 2**.

### Immune-mediated treatment response

**Figure 3:**
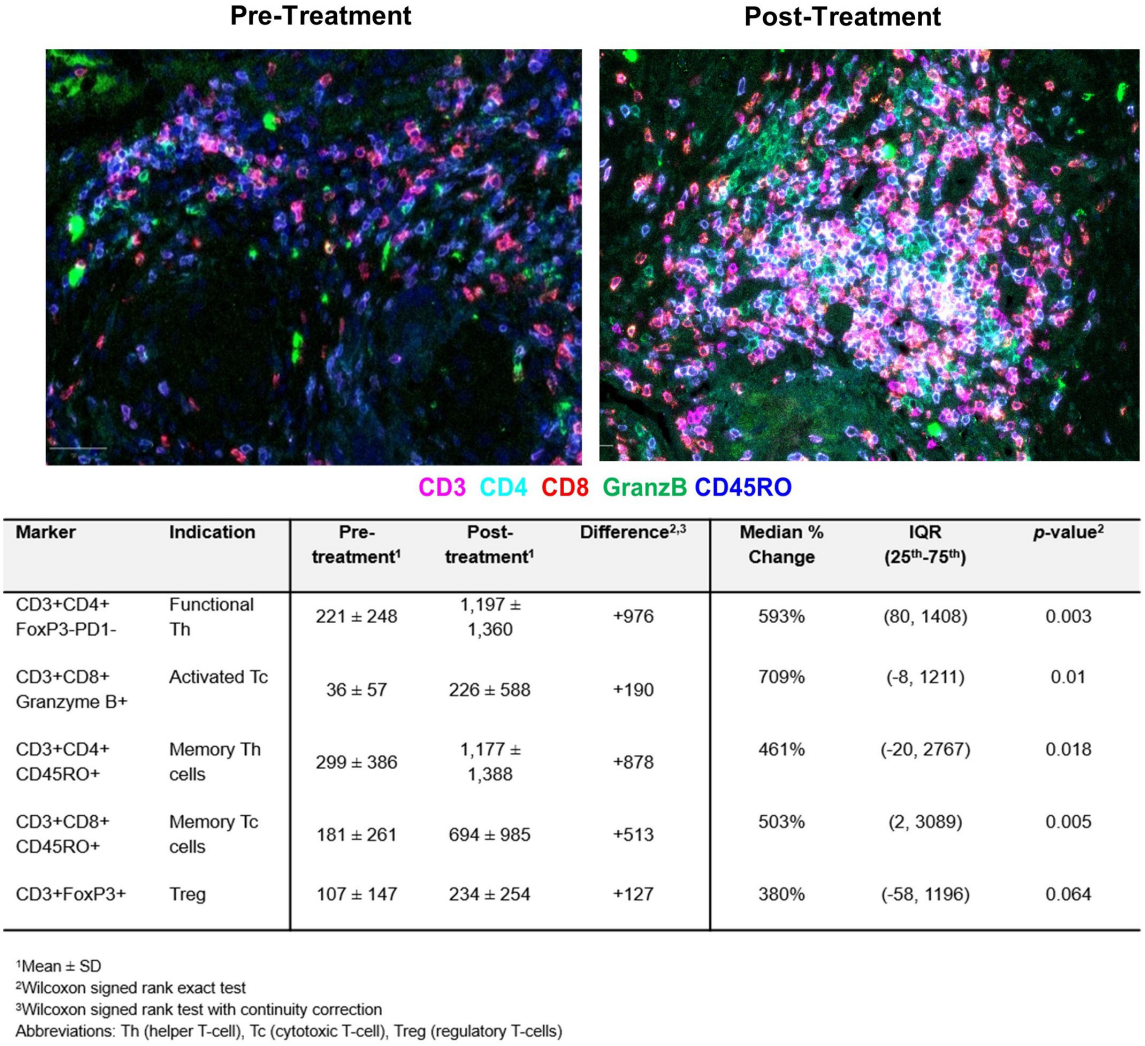
Tumor-infiltrating lymphocyte (TILs) counts pre- and post-treatment assessed by quantitative multiplex immunofluorescence analysis. The results are expressed as the average cell density in 1 mm^2^ (cells/ mm^2^). A significant increase in functional helper and cytotoxic T-cells and memory T-cells is observed.

To assess the antitumor immune activity, immune cell composition in the tumor bed from paired pre- and post-treatment tissues was evaluated in all fifteen patients by qmIF. As shown in **Figure 3**, the most striking findings were a 709% increase in cytotoxic T-cells (*p*=0.01) and a 593% increase in helper T-cells (*p*=0.003). 503% and 461% increase in memory cytotoxic and helper T-cells, respectively, were also noted. Although not significant, an increase in regulatory T-cells by 380% was detected. Spearman’s rank correlation assessed the contribution of changes in various tumor-infiltrating lymphocytes (TILs) to the reduction in tumor cell count (**Table 3**). Both activated cytotoxic and helper T-cells significantly correlated with the reduction in tumor cell count (R=0.55; *p*=0.036 and R=0.54; *p*=0.039, respectively), but not the memory T-cells.

**Table 3:** Correlation between median percent change in TILs and reduction in tumor cell count pre- and post-treatment assessed by quantitative multiplex immunofluorescence analysis.

T-cells	R¹	95% CI¹	*p*-value^2^
Functional Th-Cells	0.54	(0.06,0.84)	0.039
Activated Tc	0.55	(-0.06,0.97)	0.036
Memory Th	0.45	(-0.13,0.83)	0.097
Memory Tc	0.49	(-0.04,0.87)	0.065

^1^R = Rho, CI = Confidence Interval1R = Rho, CI = Confidence Interval

^2^Spearman’s rank correlation

Th, helper T-cell; Tc, cytotoxic T-cell; Treg, regulatory T-cells.

**Figure 4:**
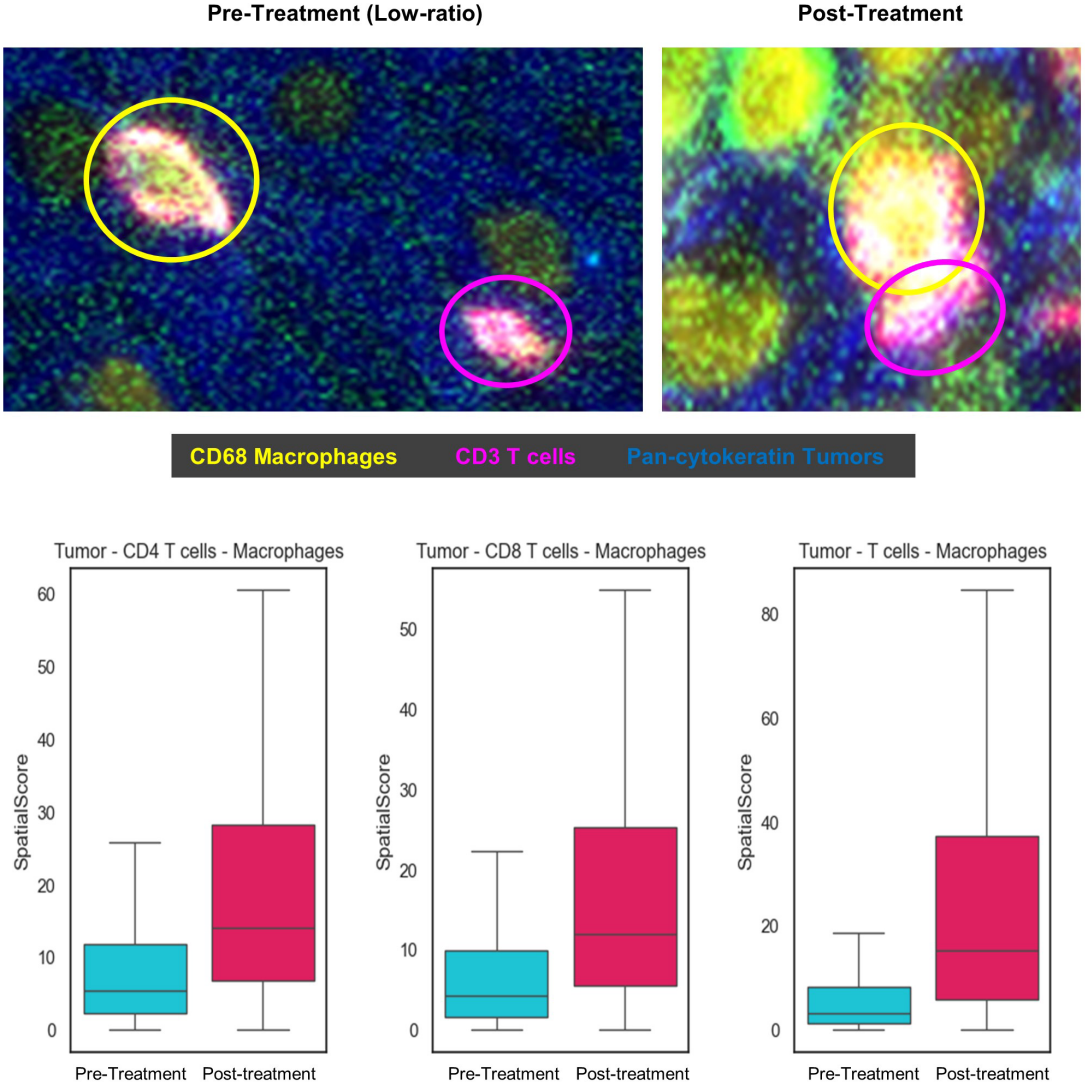
Spatial Score between tumor, helper and cytotoxic T-cells, and macrophage, pre- and post-treatment. The spatial score is an average of minimal distance between each cell type and its nearest other cell types. A low ratio reflects T-cells in proximity with the tumor cells relative to macrophages and a high ratio is T-cells in proximity with the macrophages relative to tumor cells.

The spatial topography of helper and cytotoxic T-cells to tumor cells relative to the proximity of these T-cells to macrophages was measured to explore their spatial relationships. In the pre-treatment tissue, a lower ratio (helper and cytotoxic T-cells in close proximity to tumor than to macrophage) was observed. After the treatment, the helper and cytotoxic T-cells were in closer proximity to macrophage than tumors resulting in a higher spatial ratio as shown in **Figure 4**. The helper and cytotoxic T-cells and all T-cells were closer in distance to tumor cells before the therapy and shifted closer to macrophages post-therapy.

### PD-L1 expression

PD-L1 expression analysis was performed using the quantified data obtained from qmIF. **Supplementary Figure 2** shows a heatmap of the total tumor cell count, total number of tumor and immune cells expressing PD-L1, and PD-L1+ tumor and macrophage count within the tumor bed. As shown in **Table 4**, there was no change in the median PD-L1+ tumor cell numbers post-treatment. However, there was a slight reduction in PD-L1+ macrophage cell counts (*p*=0.041), while the median percent change showed an increase (158%, *p*=0.007).

**Table 4:** The median percentage change of PD-L1+ tumor cells and macrophages by quantitative multiplex immunofluorescence analysis.

Marker	Indication	Pre-treatment^1^	Post-treatment^1^	Difference(*p*-value)^2,3^	Median % Change	IQR (25^th^-75^th^)	*p*-value^2^
CK+PD-L1+	Tumor-induced immune suppression	146 ± 316	126 ± 431	+976 (0.780)	0%	(64, 208)	0.093^1^
CD68+ PD-L1+	Macrophage-mediated immune suppression	177 ± 551	124 ± 231	-53 (0.041)	158%	(14, 162427)	0.007^2^

^1^Wilcoxon signed rank test with continuity correction.

^2^Wilcoxon signed rank exact test The results are expressed as the average cell density in 1 mm^2^ (cells/ mm^2^).

TPS accounting for proportions of PD-L1+ viable tumor cells and CPS inclusive of PD-L1+ tumor and immune cells correlated with irRVT and cancer recurrence is shown in **Table 5**. TPS positivity (≥1%) was found in 7 patients pre-treatment and 7 patients post-treatment. At baseline, CPS <1% was observed in 7 patients, 1-9% in 5 patients, 10-19% in 0 patients, and >20% in 3 patients. Following imiquimod therapy, CPS <1% was observed in 2 patients, 1-9% in 7 patients, 10-19% in 0 patients, and >20% in 6 patients. Pre-treatment TPS and CPS were <5% (range 0-3%) for all nine patients with irMPR. Interestingly, one patient who had recurrent cancer exhibited the highest pre-treatment CPS of 102%.

**Table 5:** Predictors of treatment response and recurrence-free survival. Tumor proportion score (TPS) and combined positivity score (CPS) are calculated based on PD-L1 expression

Pre-Treatment		Post-Treatment		%irRVT	Recurrence
TPS	CPS	TPS	CPS		
0%	0%	0%	1%	0	No
0%	1%	0%	9811%	0	No
1%	1%	12%	28%	5	No
0%	0%	0%	3%	5	No
0%	0%	0%	1%	5	No
0%	0%	0%	0%	10	No
1%	1%	1%	9%	10	No
0%	0%	1%	5%	10	No
2%	3%	3%	33%	10	No
43%	45%	1%	8%	25	No
0%	0%	0%	0%	35	No
21%	33%	53%	77%	40	No
0%	0%	0%	28%	40	No
15%	102%	0%	29%	45	Yes
1%	3%	1%	5%	65	No

The CPS accounting for PD-L1 expression of both tumor and immune cells have prognostic value in terms of treatment response and recurrence-free survival. The pre-treatment CPS correlated with the percent residual viable tumor (%irRVT) and one-year cancer recurrence.

Color shading represents pre- and post-treatment TPS and CPS of one patient who had cancer recurrence; this patient had the highest pre-treatment CPS.

### Recurrence-free survival

Fourteen patients had sufficient follow-up and were evaluable for one-year RFS via electronic chart review. Median follow-up was 17 months (95% CI = 16 months - not reached) based on the reverse KM approach. Following neoadjuvant imiquimod therapy and surgical resection, 13 (93%) of 14 patients had recurrence-free 1-year survival. One patient developed a recurrent OSCC immediately adjacent to the previous tumor site approximately six months after the surgery despite negative surgical margins. The lesion was subsequently resected, and the patient had no further evidence of disease. The duration of follow-up and the one-year recurrence-free survival status of individual patients are shown in **Table 6**.

**Table 6:** Duration of follow-up from the time of surgery and one-year cancer recurrence status.

Patient Number	Recurrence follow-up (months)	One-year RFS
1	29.7	RF
2	9.6	NE
3	5.6	R
4	12.0	RF
5	17.4	RF
6	17.0	RF
7	15.6	RF
8	14.7	RF
9	16.6	RF
10	19.3	RF
11	12.0	RF
12	30.3	RF
13	21.5	RF
14	28.3	RF
15	29.5	RF

RF, Recurrence free; R, Recurrence; RFS, Recurrence-free survival.

## Discussion

Neoadjuvant use of imiquimod prior to surgical resection of early-stage oral cancer was feasible and well tolerated. The primary efficacy endpoint of >50% reduction in tumor cell count was met in 60% of patients, and the secondary safety endpoint in all participants. With direct access to the tumors, the clinical tumor size was obtained by measuring the longest perpendicular bidirectional size of the clinically visible lesion instead of using response evaluation criteria in solid tumors (RECIST). Immune-related pathologic response criteria (irPRC) is a standardized, universal scoring system for assessing pathologic response to immunotherapy, inclusive of immune-mediated tumor regression (19, 20, 28). Similar to RECIST, the measure of clinical size is limited in accurately assessing the percent residual viable tumor (irRVT) from the tumor bed, resulting in discrepancies in response rates between clinical and pathologic assessments (19). In attempts to eliminate interobserver variability during the pathologic analysis and scoring, the tumor bed area was macrodissected, and tumor cells were quantified using qmIF. However, an increase in tumor cell counts despite the reduction in clinical tumor size was observed in three patients. This may be explained by the biopsy taken from a less dense tumor area compared to the most tumor-populated area selected from the surgical tumor specimen for analysis, a limiting factor for tumor cell quantification-based therapy response assessment. irRVT of these three patients demonstrated immune-mediated tumor regression. Hence, irPRC method complements the qmIF-based tumor cell quantification strategy for assessing antitumor response to immunotherapy.

In our study, all patients received scheduled surgery within 29-42 days of initiating imiquimod therapy, demonstrating the feasibility of neoadjuvant imiquimod therapy without delay in surgery. Significant local progression and lymph nodal metastasis are found with time-to-treatment initiation >70 days (29). Hence, minimizing the time lapse between initial diagnosis and definitive surgery was crucial in our pilot neoadjuvant trial. Imiquimod was well-tolerated with no life-threatening (grade 4) toxicity. One-year recurrence-free survival was 93% in fourteen evaluable patients. For early-stage OSCC, one-year recurrence-free survival rate is 80% for those treated with surgery and neck dissection (30). Although statistical analysis could not be conducted with a limited sample size, our data suggests a recurrence-free survival benefit of neoadjuvant imiquimod therapy.

Imiquimod induces a marked increase in tumor-infiltrating cytotoxic and helper T-cells, which exerts antitumor activity (31, 32). Indeed, functional cytotoxic and helper T-cells increased by ~500% and significantly correlated with a reduction in viable tumor cell count. Our finding concordances with others reporting a correlation between increase in TILs and pathologic response in oral cancer patients receiving neoadjuvant immunotherapy (33–35). There were 503% and 461% increase in memory cytotoxic and helper T-cells. An increase in memory T-cells correlated with the absence of signs of early metastatic invasion, a less advanced pathological stage, and improved survival (36). In our study, an unexpected increase in regulatory T-cells (Tregs) was observed, although not significant. Imiquimod, similar to tremelimumab (anti-CTLA-4 antibody IgG2 isotype), may replenish effector and memory TILs without influencing the proportion of Tregs (37).

PD-L1 expression on tumor cells, TILs, and macrophages is associated with treatment response and resistance to immune-checkpoint inhibitors (32). Pre-treatment PD-L1 expression by tumor cells was low. This is consistent with others reporting 24-35% PD-L1 positivity in OSCC tumor tissue samples (38). A minimal change in the number of PD-L1+ tumor cells was observed after the treatment. Quantification of PD-L1+ macrophages showed a slight decrease in cell count but a median increase of 158% post-therapy. Unlike immune checkpoint inhibitors, imiquimod may not participate in modulating the PD-L1 pathway.

It is important to note that immune cell deprivation in TME is considered more detrimental in dampening antitumor activity than high PD-L1 expression (39). Imiquimod activates macrophage-mediated tumor cell killing by effectively transforming immunosuppressive M2-tumor associated macrophages (TAMs) to immunosusceptible M1-TAMs (31, 32). In our study, spatial analysis showed both helper and cytotoxic T-cells moving closer to macrophage after imiquimod therapy. This conflicts with immune checkpoint inhibitor studies reporting closer proximity between tumor and T-cells compared to macrophage and T-cells post-therapy, corresponding to improved therapeutic response (26, 27). TAM reprogramming induces macrophage-mediated killing of cancer cells, and recruitment and activation of innate and adaptive lymphoid cells (32). Taken together, imiquimod may induce a strong and coordinated infiltration of activated TILs in TME by functional reprograming of TAMs, a crucial antitumor activity that may overcome PD-L1-mediated immune evasion. Further study is needed to fully understand the complex mechanistic and spatial interactions between cell types and their impact on effector cell function.

Nevertheless, PD-L1 expression may be a marker of treatment response and recurrence-free survival. The combined positive score (CPS) accounting for PD-L1 expression of both tumor and immune cells is more prognostically valuable than the tumor proportion score (TPS), which measures the expression of PD-L1 on tumor cells only (39). In our study, pre-treatment TPS and CPS correlated with the treatment response (irRVT). One patient who had a cancer recurrence had the highest pre-treatment CPS, indicating that pre-treatment CPS may have prognostic significance.

Imiquimod is available on the market as a 5% cream (Aldara^®^) and Food and Drug Administration (FDA)-approved for topical therapy for anogenital warts, actinic keratosis, and superficial basal cell carcinomas (40). The off-label use of imiquimod has demonstrated efficacy in treating oral dysplasia and OSCC (6, 7, 40). In this study, patients applied one packet of imiquimod daily (containing 12.5 mg of imiquimod) for 28 days. The dosing schedule is equivalent to three times per week for nine weeks, less than the recommended dose of three times per week for 16 weeks for skin carcinomas (41). The time for absorption of drugs into the oral mucosa is short, and any residual medication is washed with saliva after 5 to 10 minutes of application (40, 42). Residual imiquimod mixed in saliva can irritate the oropharynx, resulting in pharyngitis. The composite microneedle patch, consisting of 100 dissolvable microneedles with drug-loaded tips and a backing layer, has been designed to improve the oral transmucosal drug delivery efficiency (43). Such drug delivery modality will be explored in future imiquimod clinical trials. A tablet form of TLR-7 (TQ-A3334) and intratumoral injection (LHC165) are also currently being explored (18, 44). Assuming the extent of absorption of imiquimod through the oral mucosa is similar to that of the dermis (~ 0.6% per application and terminal half-life of 20 hours) (41), the smaller dose used in our study compared to that recommended for skin cancer may have contributed to a lack of more serious systemic adverse events, such as flu-like symptoms.

This was a single-arm study with no control arm and limited enrollees. Transmucosal administration of imiquimod may be affected by saliva flow, leading to premature swallowing of the drug before being sufficiently absorbed by the mucosa, resulting in ineffective drug delivery. The immune biomarker analysis was limited to tumor tissue and lacked correlative measures with the peripheral blood immune reactivity. While the study was initially intended to be single-institutional, due to the relocation of the principal investigator, patients were enrolled from two geographically distinct areas. No sub-analysis by region was performed due to the small target sample size.

This is the first neoadjuvant clinical trial for early-stage oral cancer patients utilizing a topical immunotherapeutic agent. A significant reduction in clinical tumor size and pathologic response was achieved in patients. The tumor microenvironment demonstrated an active antitumor immune response following the treatment. A larger randomized trial is necessary to assess treatment efficacy.

## Data Availability

The original contributions presented in the study are included in the article/**Supplementary Material**. Further inquiries can be directed to the corresponding author.
