# Supplementary material for: Pilot clinical trial of neoadjuvant toll-like receptor 7 agonist (Imiquimod) immunotherapy in early-stage oral squamous cell carcinoma
Source: Front Immunol. 2025 Jan 27;16:1530262. doi: 10.3389/fimmu.2025.1530262 (PMC11807971; doi:10.3389/fimmu.2025.1530262)
Supplement: Supplementary file 1 [file DataSheet1.docx]

**Supplementary Materials**

**Supplementary Table 1**. Therapy-related adverse events (Common Terminology Criteria for Adverse Events Version 5.0) of individual participants.

| **Patients** | **Adverse Events** | **CTCAE Grade** |  |
| --- | --- | --- | --- |
| 1 | Oral mucositis | 2 | 1= asymptomatic or mild |
|  | Fatigue | 1 | 2= moderate, intervention indicated |
|  | Oral pain/Sore throat | 1 | 3= severe, interfering with oral intake |
| 2 | Oral mucositis | 2 |  |
|  | Fatigue | 2 |  |
|  | Oral pain/Sore throat | 2 |  |
| 3 | Oral pain/Sore throat | 2 |  |
| 4 | Oral pain/Sore throat | 2 |  |
| 5 | Oral mucositis | 1 |  |
|  | Oral pain/Sore throat | 1 |  |
|  | Nausea | 1 |  |
| 6 | none | - |  |
| 7 | Oral mucositis | 2 |  |
| 8 | Oral mucositis | 1 |  |
|  | Oral pain/Sore throat | 3 |  |
| 9 | Oral mucositis | 1 |  |
|  | Oral hemorrhage | 2 |  |
| 10 | Oral mucositis | 2 |  |
| 11 | Oral mucositis | 2 |  |
| 12 | Oral mucositis | 3 |  |
| 13 | Oral mucositis | 1 |  |
| 14 | Oral mucositis | 2 |  |
| 15 | Oral mucositis | 1 |  |

**Supplementary Figure 1.** Clinical and pathologic features of therapy response. (A) Pre-treatment image of a TNM Stage II squamous cell carcinoma of the right lateral tongue. (B) Twenty-eight days following imiquimod topical therapy demonstrating smoothening of the tumor surface. (C) Photomicrograph of the pre-treatment biopsy tissue (40x) and (D) post-treatment surgical tissue (40x); orange boxes represent the tumor bed macrodissected for multiplex immunofluorescence analysis. (E) High-power view showing dense tumor infiltration at baseline (400x) and (F) High-power view showing a reduction in the number and density of tumor islands post-treatment (400x).

**Pre-treatment** **Post-treatment**

**A. B.**


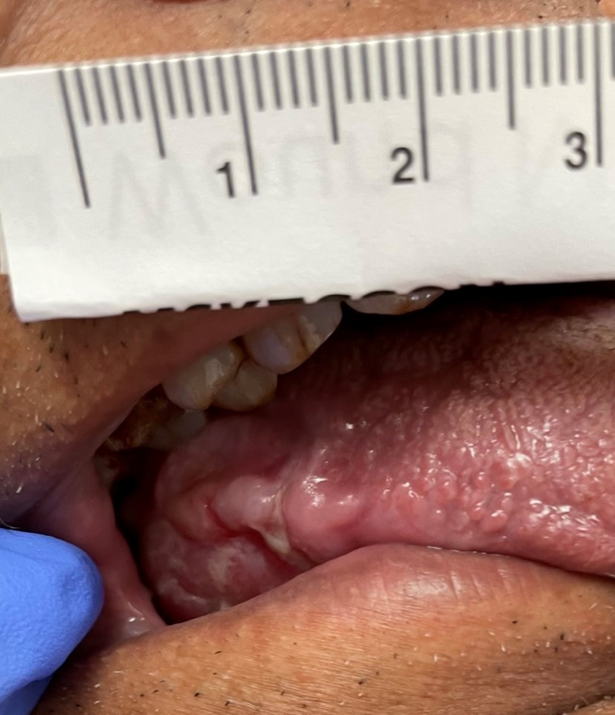

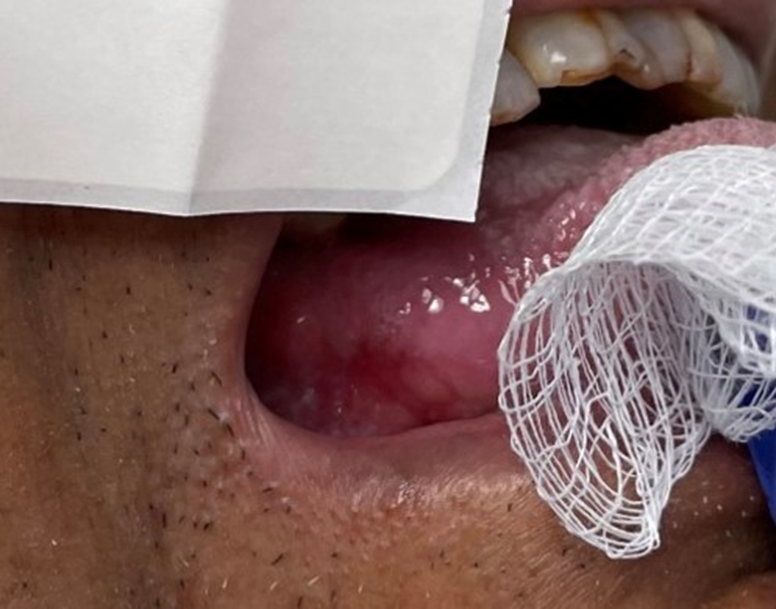


**C. D.**


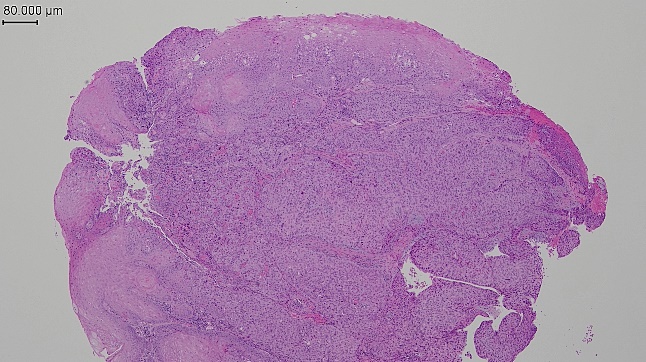

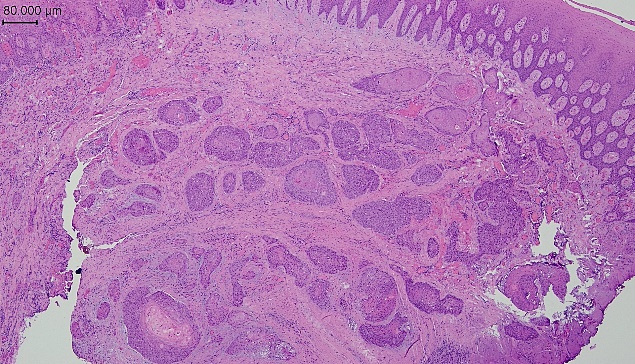


**E. F.**


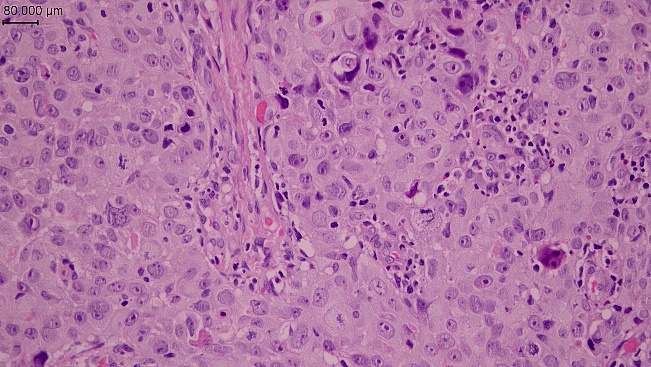

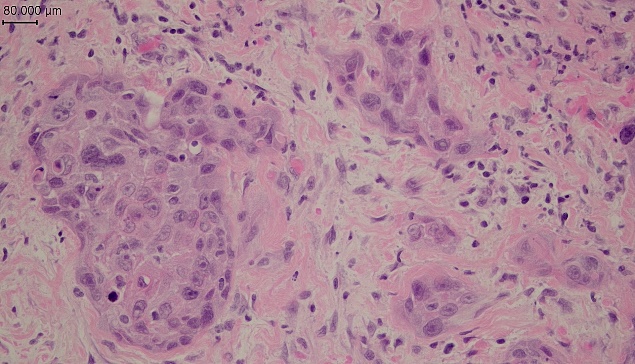


**Supplementary Table 2.** Change in clinical tumor size, tumor cell count, percent residual viable tumor, and immune-mediated pathologic response for individual patients.

| **Patient Number** | **Clinical size change (%)** | **Tumor cell count change (%)** | **irRVT (%)** | **Pathologic Response** |
| --- | --- | --- | --- | --- |
| 1 | -53.6 | -77.3 | 5 | MPR |
| 2 | -12.0 | -35.4 | 40 | PR |
| 3 | -20.0 | -75.3 | 50 | PR |
| 4 | -45.5 | -34.0 | 35 | PR |
| 5 | -31.8 | 317.0 | 45 | PR |
| 6 | -66.7 | -65.4 | 20 | PR |
| 7 | -38.9 | 82.7 | 65 | PR |
| 8 | -23.5 | -99.9 | 20 | PR |
| 9 | -33.3 | -68.3 | 50 | PR |
| 10 | 0 | 129.2 | 15 | PR |
| 11 | -50.0 | -100.0 | 0 | pCR |
| 12 | -54.5 | -78.2 | 5 | MPR |
| 13 | -54.5 | 7.2 | 40 | PR |
| 14 | -75.0 | -100.0 | 0 | pCR |
| 15 | -100 | -63.7 | 10 | MPR |

Abbreviations:

irRVT: Residual viable tumor (Based on the Immune-Related Pathologic Response Criteria)

MPR: Major pathologic response (<10% RVT)

pCR: Pathologic complete response

PR: Partial response

**Supplementary Figure 2**. Heatmap of tumor (CK), PD-L1, and PD-L1+ tumor and macrophages (CD68) cell counts assessed by quantitative multiplex immunofluorescence analysis (cells/mm^2^).

| **Pre-Treatment** | | | | **Post-Treatment** | | | |
| --- | --- | --- | --- | --- | --- | --- | --- |
| CK | PD-L1 | PD-L1+ Tumor | PD-L1+ Macrophages | CK | PD-L1 | PD-L1+ Tumor | PD-L1+ Macrophages |
| 2517.91 | 507.28 | 16.17 | 6.16 | 570.83 | 527.64 | 4.11 | 45.94 |
| 2467.23 | 50.39 | 0.00 | 1.05 | 1593.60 | 55.19 | 1.29 | 22.42 |
| 5917.93 | 29.00 | 0.00 | 1.58 | 1462.40 | 54.48 | 1.80 | 3.15 |
| 3020.77 | 303.64 | 67.92 | 32.76 | 1993.47 | 2387.40 | 52.92 | 597.55 |
| 232.29 | 5.58 | 1.68 | 0.00 | 968.77 | 183.80 | 118.87 | 147.64 |
| 1659.86 | 8.78 | 0.00 | 1.10 | 573.64 | 12.19 | 2.36 | 1.57 |
| 1064.48 | 4.14 | 0.38 | 0.00 | 1945.13 | 167.85 | 5.46 | 50.75 |
| 2853.46 | 21.01 | 3.15 | 1.05 | 0.28 | 5.65 | 0.00 | 0.28 |
| 2537.59 | 1845.22 | 1090.39 | 52.88 | 804.42 | 95.76 | 6.85 | 56.08 |
| 634.97 | 6.84 | 0.68 | 0.68 | 1455.67 | 370.69 | 13.54 | 54.15 |
| 322.92 | 12.11 | 0.00 | 0.00 | 0.00 | 1925.49 | 0.00 | 98.11 |
| 2128.36 | 187.55 | 16.80 | 57.38 | 463.00 | 31.91 | 6.49 | 18.12 |
| 2954.19 | 1257.29 | 620.71 | 351.29 | 3166.18 | 3482.41 | 1678.74 | 757.39 |
| 2459.93 | 2148.67 | 371.98 | 2142.90 | 0.00 | 2.05 | 0.00 | 0.29 |
| 2746.10 | 3.28 | 0.00 | 0.00 | 995.55 | 7.09 | 0.00 | 1.58 |

Graded color scale of cell count


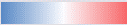


Low High
